# Customer-centric product presentations for monoclonal antibodies

**DOI:** 10.1186/s41120-022-00069-y

**Published:** 2023-01-23

**Authors:** Beate Bittner

**Affiliations:** grid.417570.0 0000 0004 0374 1269 F. Hoffmann-La Roche Ltd., Global Product Strategy - Product Optimization, Grenzacher Strasse 124, CH-4070 Basel, Switzerland

**Keywords:** Customer centricity, Parenteral, Oral, Subcutaneous, Flexible care setting, Product optimization

## Abstract

Delivering customer-centric product presentations for biotherapeutics, such as monoclonal antibodies (mAbs), represents a long-standing and paramount area of engagement for pharmaceutical scientists. Activities include improving experience with the dosing procedure, reducing drug administration-related expenditures, and ultimately shifting parenteral treatments outside of a controlled healthcare institutional setting. In times of increasingly cost-constrained markets and reinforced with the coronavirus pandemic, this discipline of “Product Optimization” in healthcare has gained momentum and changed from a nice-to-have into a must.

This review summarizes latest trends in the healthcare ecosystem that inform key strategies for developing customer-centric products, including the availability of a wider array of sustainable drug delivery options and treatment management plans that support dosing in a flexible care setting. Three disease area archetypes with varying degree of implementation of customer-centric concepts are introduced to highlight relevant market differences and similarities. Namely, rheumatoid arthritis and inflammatory bowel disease, multiple sclerosis, and oncology have been chosen due to differences in the availability of subcutaneously dosed and ready-to-use self-administration products for mAb medicines and their follow-on biologics.

Different launch scenarios are described from a manufacturer’s perspective highlighting the necessity of platform approaches. To unfold the full potential of customer-centric care, value-based healthcare provider reimbursement schemes that incentivize the efficiency of care need to be broadly implemented.

## Introduction

Over the past quarter century, biotherapeutics, such as monoclonal antibodies (mAbs), have become a prevailing novel treatment modality (Lu et al. 2020) and as such significantly contribute to both the costs (Hernandez et al. 2018) and the environmental impact (Amasawa et al. 2021) of healthcare. Inherent to their physicochemical properties, mAbs must be administered parenterally, a circumstance that can be considered inconvenient (Fernández et al. 2017) and frequently mandates more burdensome in-clinic dosing (Bohra et al. 2020).

In times of continuously growing cost pressure on healthcare and major implications of the pandemic on established medical services (Arsenault et al. 2022), any effort in minimizing the dosing complexity of parenteral administration has the potential to reduce expenditures for the drug administration procedure. For instance, if permitted by the safety profile of a biological medicine, an attempt to lessen in-clinic time during an intravenous dosing day is the provision of fast infusion regimens. This approach can improve cost-effectiveness of the treatment, as it allows more people to be treated in the clinic within a given time frame (Spadaro et al. 2017). To further facilitate intravenous dosing and to aid operators with obtaining central vascular access, new device types, such as a handheld assistive artificial intelligence-enabled, ultrasound-guided robotic device for intravenous catheterization, are currently undergoing early development (Brattain et al. 2021).

An alternative concept for optimizing hospital resource utilization that has been in the center of interest of many drug delivery researchers in recent years represents reformulating a biological medication for application via a less-invasive administration route. In 2014, Tetteh et al. (2014) developed a regression-based algorithm that included an estimate on how the administration route of biotherapeutics, including mAbs, may impact on healthcare delivery expenditures. The analysis suggests that assuming no other change (that is, in-clinic dosing, dosing frequency), subcutaneous or intramuscular administration of biologics lowers total healthcare delivery costs as compared to intravenous infusions.

A more significant step in reducing dosing regimen-related inconvenience, healthcare institutional spending and in improving affordability and access to mAb treatments is to shift dosing outside of the clinical setting (Wolfromm et al. 2017; Bittner et al. 2018; Bittner and Schmidt 2021). Here, subcutaneous at-home- and self-administration has become an established dosing regimen for mAbs with a favorable safety and tolerability profile across different disease areas (Gottlieb et al. 2016, Raffaelli et al. 2019, Van den Bemt et al. 2019, Timmermann et al. 2020, Jappe et al. 2021, Bagel et al. 2022). Especially low-volume subcutaneous injections can be self-administered by means of a variety of prefilled syringes or pen device types, thus accounting for personal priorities and capabilities (Anderson and Redondo 2011; Vermeire et al. 2018). While these automated dosing aids support convenience with an at-home dosing regimen, adherence and persistence to subcutaneous administration in an unsupervised setting varies between medical products (Tkacz et al. 2014; Nieto et al. 2021). Besides disease severity or prior experience with subcutaneous administration, also the dosing schedule can contribute to compliance issues with the prespecified application procedures (Tkacz et al. 2014).

The situation is complicated specifically for high-dose mAbs requiring comparatively large administration volumes and for mAbs dosed in combination therapy. While user preference assessments demonstrate that even in the hospital setting, high-dose subcutaneous injections with individual dose volumes between 5 and 15 milliliters (mL) are preferred over intravenous infusion regimens (Pivot et al. 2014; Rummel et al. 2017; O'Shaughnessy et al. 2021; Usmani et al. 2021), customized dosing schemes for at-home administration still remain to be developed by manufacturers in close cooperation with healthcare providers and regulators. This is particularly the case for mAbs that exhibit severe infusion-related reactions (IRRs) mainly occurring during the first dosing cycles (Rombouts et al. 2020).

To improve adherence to parenteral dosing in a non-controlled setting, the individual demands of people treated with mAbs have to be accounted for by manufacturers. In addition to interventions that address educational, behavioral or psychological barriers against complying with the dosing regimen (Remington et al. 2013), providing personalized and thus customer-centric product presentations and administration schemes has the potential to enhance adherence to parenteral home administration overall (Ridyard et al. 2016).

The term customer centricity is applied across industries highlighting that in order to achieve sustainable product offerings, satisfying the needs of “the customer” has to be the ultimate focus for any development decision (Pardo-Jaramillo et al. 2020). For medicinal products, “the customer” is classically defined as “the patient,” but increasingly the entire healthcare ecosystem is referred to using this term. Thus, in addition to people receiving treatment for a diagnosed medical condition, this ecosystem comprises professional healthcare providers and institutions, regulators, payers (Pidun et al. 2021), and ultimately society as a whole.

In the pharmaceutical industry, efforts to ameliorate the product profile of an established medicine are driven by the cross-functional discipline of “Product Optimization” (Bittner and Schmidt 2022). “Product Optimization” also referred to as “Formulation and Device Lifecycle Management” aims at improving the drug delivery profile and product presentation for a medicine that is either already on the market or in late-stage clinical development and thus at providing a more customer-centric presentation.

This review is written from a manufacturer’s perspective and summarizes key trends in the healthcare ecosystem that define customer-centric drug delivery requirements for mAbs across different therapeutic areas, illustrates approaches to obtain insights into emerging drug delivery necessities, and compares the latest developments in three distinct disease area archetypes.

## Drug delivery as treatment enabler versus as customer-centric differentiator

Drug delivery is commonly explained as the “method or process of administering a pharmaceutical compound to achieve a therapeutic effect” (Gupta and Kumar 2012; Tiwari et al. 2012). This definition predominantly refers to drug delivery technologies that enable the administration of pharmaceutical products per se. Such is particularly the case when developing formulation or device technologies for novel treatment modalities with previously unexplored physicochemical and pharmacokinetic properties. Frequently, in these instances, there is neither experience available on how a galenical formulation can impact bioavailability, safety or efficacy of the medication, nor on what would be the most preferred product profile from a customer perspective. Focus of drug delivery scientists is therefore on progressing a product presentation that achieves the required target exposure via an appropriate administration route. Especially with new and possibly disease-modifying biological molecules, technologies that go beyond treatment enablement may either have not yet been identified or are still in early development (Blanco and Gardinier 2020). Aspects of convenient dosing or efficient healthcare resource utilization are of secondary priority at this stage. Consequently, any formulation and device optimization that would delay initial molecule launch and thus availability of the medication for customers is typically introduced as a lifecycle management (Bittner and Schmidt 2022).

“Customer-centric” drug delivery, as defined in this article, becomes an important aspect for indications with a variety of more mature medications with similar physicochemical properties available from a given compound class. Here, treatment enabling technologies have been established over time (Shams et al. 2021). With emerging customer insights on needs for improving the product presentation, drug delivery efforts predominantly focus on differentiating the medication with a more convenient and cost-efficient profile (Roy et al. 2021; Schreiber et al. 2022).

In addition to achieving a user-oriented drug delivery profile, manufacturers engage in offering medicines with overall more sustainable presentations. Activities entail the implementation of measures to reduce drug wastage via more economic dosing regimens and supply chain concepts (Hendrikx et al. 2017; Tat and Heydari 2021).

Over time, and on the basis of progressively established platform technologies, this initially stepwise approach is emerging into a situation where novel products are available with the best feasible drug delivery profile from initial molecule launch onwards.

## Healthcare trends that define customer-centric mAb product presentations

Today, people diagnosed with a chronic condition are increasingly well informed about particularities of their disease and willing to actively participate in the design of healthcare processes (Longtin et al. 2010). Resulting customer-centric concepts commonly include aspects of disease self-management (Holmes et al. 2019) and as such mandate measures that allow unsupervised parenteral dosing. In this case, providers, in order to support dosing in a remote setting, require reassurance that high-quality disease monitoring and adherence to treatment procedures is maintained. Assuming compliance and adequate support services are guaranteed for parenteral administration outside of a healthcare institution, the underlying societal benefits of at-home- and particularly the economic benefits of self-administration (Franken et al. 2020) are pivotal elements of value-based healthcare (Dainty et al. 2018, Teisberg et al. 2020).

From a regulator’s perspective, mAbs suitable for home administration must possess an appropriate safety profile and must be available in a product presentation that supports unsupervised administration (Bittner and Schmidt 2022). As data derived from registrational clinical trial investigations alone may not exhaustively reveal feasibility of a flexible care setting, additional sources of information are being considered. In assessing the risk benefit of a medicinal product, regulators progressively encourage manufacturers to actively implement real-world data (RWD) and real-world evidence (RWE) during development processes and to share “patient-provided information” for evaluation as part of drug and medical device applications (United Stated Food and Drug Administration 2018). The United States Food and Drug Administration (FDA) defines RWD as “data relating to patient health status and/or the delivery of health care routinely collected from a variety of sources” and RWE as “the clinical evidence about the usage and potential benefits or risks of a medical product derived from analysis of RWD” (United States Food and Drug Administration 2022b). According to the FDA, RWD sources can include registries, collections of electronic health records, or administrative and medical claims databases. Besides prescription information, medical diagnoses, bills submitted to payer organizations, costs, charges and reimbursement amounts, such databases also include insights into procedures and treatments performed (Rocco et al. 2017; Park and Lee 2021). Globally, clinical trial results are therefore supplemented by RWD and RWE (Hiramatsu et al. 2021). This evidence also serves as a source for estimating the impact of drug delivery modalities on healthcare resource utilization (Stearns et al. 2019).

Likewise, the application of robust RWE to supplement experimental evidence in coverage decisions is being considered globally (Facey et al. 2020). In a literature review on the US healthcare system conducted by Hampson et al. (2018), comparative clinical effectiveness and network meta-analysis for quantitative indirect comparisons were identified as pivotal sources for initial payer coverage and Heath Technology Assessment decisions. Here, “patient-reported data” may be used as a complimentary data source. For reassessments, that is reconsidering coverage, formulary placement or payment terms, as well as in the context of outcomes-based contracting, RWE can play a role in further defining the clinical or economic value of an intervention, beyond the evidence generated in the clinical trial setting. Notably, in their review, a number of challenges associated with the use of RWE for healthcare payment decisions were identified. These include aspects of reporting bias, incomplete data, lack of universally accepted methodological standards, lack of investigator expertise, or obsolete evidence hierarchies. Similar findings were made in a US payer interview conducted by Timbie et al. (2021). The evaluation identified the evidence from rigorous clinical trials as a prioritized source for assessing efficacy and short-term safety findings. Some payers, however, “felt that RWE was particularly helpful when the long-term durability of devices or rare adverse events were key considerations in coverage decisions”.

**Fig. 1:**
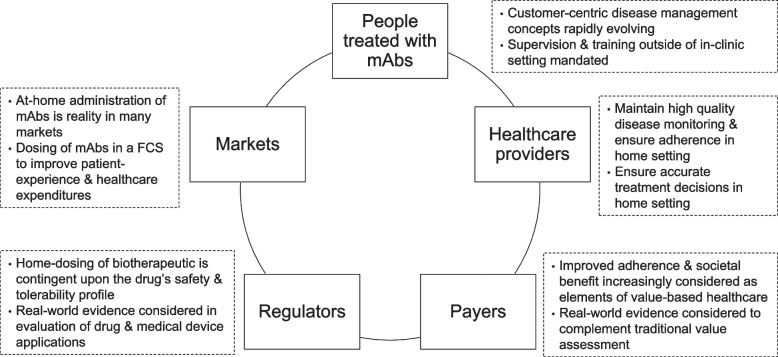
Key healthcare trends that inform customer-centric drug delivery needs for monoclonal antibodies. Need for technologies that enable dosing and data collection in a flexible care setting (FCS)

Figure 1 highlights key healthcare trends that inform customer-centric drug delivery needs for monoclonal antibodies. Insights reveal the need for technologies that enable dosing and data collection in a flexible care setting. Here, contingent upon the medication’s safety and tolerability, medication administration may take place in the clinic, a physician’s office, a community or infusion center or in the patient’s home (Bittner and Schmidt 2021).

## Customer-centric product presentations that enable dosing in a flexible care setting

Reliable application of biotherapeutics in a flexible care setting depends on adequate user training and education and mandates treatment initiation under professional supervision (Highlights of Prescribing Information (HPI) Humira®, Hizentra®, Kesimpta®: United Stated Food and Drug Administration 2022a). Establishing convenient drug delivery schemes and electronic data capturing tools permit physicians to make accurate treatment decisions while their patient is dosed outside of a controlled environment (Eun-Young 2017; El-Sappagh et al. 2019; Sebastian et al. 2019). Especially, if home dosing is facilitated with mechanisms to collect “patient-provided information,” such data could have the potential to reduce payer’s uncertainty around adherence and to complement value-based reimbursement models.

Beyond professional support services and electronic data capturing and monitoring tools, drug delivery improvements that reduce supply chain complexity, permit simple and intuitive drug administration, and facilitate medication storage and disposal represent a crucial element of pharmaceutical research and development.

**Fig. 2:**
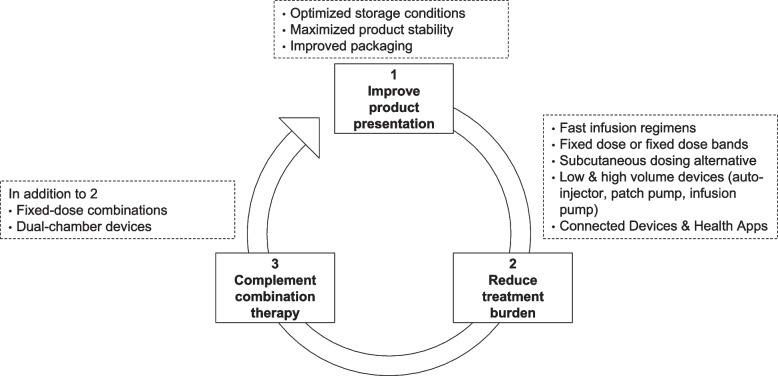
Drug delivery improvements for mAbs that reduce dosing complexity and enable dosing and data collection in a flexible care setting

Figure 2 illustrates drug delivery improvements for mAbs that reduce dosing complexity and enable dosing and data collection in a flexible care setting. Aspects include (1) improving the product presentation, (2) reducing the overall burden of parenteral drug administration, and (3) complementing combination therapy. In this order, product optimizations are expected to possess increasing potential to facilitate remote parenteral care.

Attempts in advancing the product presentation entail optimizing storage conditions and shelf-life of the medication (Kuzman et al. 2021), as well as improving its packaging to account for an easy to store and to handle offering (Zadbuke et al. 2013). Product optimizations that lessen the burden associated with parenteral administration of biotherapeutics comprise fast intravenous infusion regimens (Al Zahrani et al. 2009), fixed dosing regimens instead of body size-adjusted dosing (Egorin 2003), subcutaneous dosing alternatives to more invasive intravenous infusions (Bittner et al. 2018), or automated injection devices (Vijayaraghavan 2020). Connected devices and accompanying health apps are being implemented to support adherence in an out-patient setting and can be utilized to collect RWE on possible adverse events and share it back with the treating physician (Bittner et al. 2019). Drug delivery improvements to complement combination therapy involve the development of fixed-dose combinations with two or more mAbs co-formulated in the same dosing vehicle, or dual chamber bags (Allmendinger 2021). Details on underlying concepts and scientific nonclinical and clinical development approaches have been summarized previously and are therefore off-scope for this article (Bittner and Schmidt 2022).

## Gaining insights into customer needs for product optimization of biological medicines

To inform investments into customer-centric product optimizations for existing medicines, manufacturers regularly collect insights on possible challenges with their medications’ drug delivery profile. For marketed mAbs or for biologics in development in an indication with similar treatment modalities already employed, information on how the product might be improved is typically already available from secondary sources and thus can be estimated based on existing analogues. Notably, customers’ desires evolve with the maturity of a market based on emerging sophistication of drug delivery technologies and comparisons with other indications with biotherapeutics with similar drug delivery requirements. The journey of product optimizations is therefore flexible and adaptable to change at all times.

An understanding of user and provider preferences for one over another drug delivery methodology is gained either as part of pivotal registrational clinical trials or in smaller dedicated usability studies (Li and Easton 2018). Here, electronic apps with dosing reminders and tools to collect “patient-reported outcomes” in the home setting (El Emam et al. 2009) provide early insights into possible challenges and opportunities of a product optimization. Additionally, treatment satisfaction and quality-of-life surveys (Kempton et al. 2021), as well as time and motion studies (De Cock et al. 2014; Pivot et al. 2014) that assess the impact of the drug administration procedure on the efficiency of dosing in a prespecified setting represent an appreciable source of information.

For marketed medications and relevant reference products with a similar drug delivery profile in other indications, insights into the practicality of the drug delivery profile are frequently derived from direct user or provider feedback to the manufacturer of a medicinal product. This encompasses anecdotal reports on challenges with the drug administration instructions and at times even suggestions on how to improve these. Input is additionally tracked via established complaint management processes (Hake et al. 2019) in which customer feedback is collected and reviewed systematically over time. Verifiable customer co-creation (Adelman et al. 2019; Peng et al. 2022) may be realized via satisfaction surveys or customer workshops designing the most appropriate drug delivery system for a drug and as part of well-defined human factor trials (Lageat et al. 2021).

## Key developments in providing customer-centric product presentations for mAbs across different disease areas

### Disease area archetypes

The availability of product presentations and dosing regimens that support administration of mAbs in a flexible care setting varies considerably across indications. Three different disease area archetypes have been selected to describe the status and key developments on the journey to increasing customer centricity of mAb medications. Table 1 illustrates the relevant attributes of these different markets. Disease areas comprise rheumatoid arthritis (RA) and inflammatory bowel disease (IBD), multiple sclerosis (MS), and oncology (ONC).

**Table 1:** Key attributes of selected disease area archetypes; considers marketed mAb profiles only

Disease area archetype	mAb safety & tolerability profile	mAb drug delivery profile	Market maturity (injectables)
1 RA/IBD	mAbs exhibit sufficient tolerability to allow unsupervised administration outside of a controlled healthcare environment^a^	Predominantly SC formulations for self-administration Low dosing volumes (below 2 mL) Ready-to-use PFS & autoinjector/pen devices	Several mAbs & other biologics with different & overlapping indications available A number of mAbs & other biologics also used in other indications Biosimilars for some mAbs & other biologics available
2 MS	mAb-dependent safety profiles; unsupervised and supervised administration outside of a controlled healthcare environment^a^	Predominantly IV formulations for HCP-assisted administration 1 mAb^b^ with SC formulations for self-administration (dosing volume of 0.4 mL; ready-to-use PFS & autoinjector device)	Few mAbs with overlapping indications Biosimilars for other biologics available No mAb biosimilar marketed
3 ONC	mAb-dependent safety profiles; currently no unsupervised administration outside of a controlled healthcare environment	Predominantly IV formulations for HCP-assisted administration 4 mAbs^c^ with high-volume SC formulations for HCP-assisted administration (volumes of 5 to 15 mL; vial presentations) 1 SC mAb FDC^d^ 1 IV mAb FDC^e^	A variety mAbs with different & overlapping indications available Biosimilars for some mAbs available

*FDC* fixed-dose combination, *HCP* healthcare provider, *IBD* inflammatory bowel disease, *IV* intravenous, *mAb* monoclonal antibody, *mL* milliliters, *MS* multiple sclerosis, *ONC *oncology, *RA* rheumatoid arthritis, *SC* subcutaneous

^a^Following treatment initiation under supervision and training of a HCP

^b^Ofatumumab

^c^Trastuzumab, rituximab, daratumumab, pertuzumab + trastuzumab

^d^Pertuzumab + trastuzumab

^e^Nivolumab + relatlimab

### Disease area archetype 1 (rheumatoid arthritis and inflammatory bowel disease)—mature markets with a variety of established mAb treatments and corresponding follow-on biologics available for self-administration

Disease area archetype 1 encompasses RA and IBD, representing mature subcutaneous self-administration markets with established originator mAbs and other long-standing biological treatments and corresponding biosimilars either already approved or in development (De Figueiredo et al. 2021; Findeisen et al. 2021; Radu and Bungau 2021). Authorized mAb treatments for both RA and IBD include the tumor necrosis factor-α inhibitors infliximab, adalimumab, golimumab, and certolizumab pegol. The B-cell-depleting therapy rituximab and the interleukin-6 receptor antagonists tocilizumab and sarilumab are indicated for the treatment of RA (Senolt 2019). The interleukin-12 and interleukin-23 inhibitor ustekinumab, the α4β7 integrin antagonist vedolizumab, and the α4 integrin antagonist natalizumab are authorized for the treatment of IBD (Mao et al. 2018; Wyant et al. 2016).

In 1998, the chimeric mAb infliximab was the first tumor necrosis factor-α inhibitor authorized by the FDA for the treatment of Crohn’s disease (CD) (Melsheimer et al. 2019). Branded infliximab was and is still solely available with an intravenous dosing regimen as a powder for reconstitution (HPI Remicade®: United Stated Food and Drug Administration 2022a). The first fully human recombinant immunoglobulin G1 mAb in RA, adalimumab, was already introduced with a subcutaneous dosing regimen at initial product approval in 2002 (Marušić and Klemenčić 2018). The majority of branded injectables in the field are available with either both an intravenous and a subcutaneous formulation (golimumab, tocilizumab, ustekinumab, vedolizumab; HPIs Simponi®, Simponi Aria® Actemra®, Stelara®, Entyvio®: United Stated Food and Drug Administration 2022a, SMPC Entyvio®: European Medicines Agency 2021) or a subcutaneous formulation only (adalimumab, certolizumab pegol, sarilumab; HPIs Humira®, Cimzia®, Kevzara®: United Stated Food and Drug Administration 2022a). The fixed-dose regimens omit the need for body size-normalized dose calculation. Subcutaneous injection volumes do not exceed 2 mL and dosing frequencies range from weekly to every 4 weeks. The subcutaneous administration regimen for ustekinumab consists of an intravenous loading dose followed by every 8 weeks subcutaneous maintenance doses. Ready-to-use prefilled syringes or pen devices provide users with different dosing alternatives as per their personal requirements. Branded natalizumab, infliximab, and rituximab can only be given intravenously with maintenance dosing frequencies between every 4 weeks and every 6 months (HPIs Tysabri®, Remicade®, Rituxan®: United Stated Food and Drug Administration 2022a). These infusion regimens represent an alternative for people who prefer intravenous over subcutaneous dosing (Allen et al. 2010) or value a lower treatment frequency independent of the administration route (Huynh et al. 2014). In the clinic, less frequent dosing can be a contributor to a more convenient and cost-efficient treatment management scheme (Tetteh and Morris 2014), especially if medical examinations can be combined with a dosing day. Most notably, the fact that a variety of injectable medications are approved both for the treatment of RA and IBD increases healthcare provider’s general familiarity with an injection device type and offers the possibility for leveraging learnings on challenges with the injections (Chilton and Collett 2008; Domańska et al. 2017; Gely et al. 2019) across indications. Table 2 summarizes the mAb presentations authorized for the treatment of adults diagnosed with RA or IBD (CD and ulcerative colitis (UC)).

**Table 2:** mAbs authorized for the treatment of RA or IBD (CD and UC) in the US (up until September 2022): administration routes, dosing regimens and product presentations (adult indications)

**Indication**	**mAb**	**Administration route and dosing regimen**^a^		**IV product presentations**^a^	**SC product presentations**^a^
		**IV**	**SC**		
RA	Rituximab	2*1000 mg separated by 2 weeks (one course) every 24 weeks or based on clinical evaluation (not sooner than every 16 weeks)	-^b^	100 mg/10 mL, 500 mg/50 mL in single-dose vials	-^b^
	Tocilizumab	4 mg/kg q4w followed by 8 mg/kg q4w based on clinical response	Patients < 100 kg: 162 mg q2w, followed by increase to q1w based on clinical response Patients ≥ 100 kg: 162 mg q1w	80 mg/4 mL, 200 mg/10 mL, 400 mg/20 mL in single-dose vials	162 mg/0.9 mL in single-dose prefilled syringe or single-dose prefilled autoinjector
	Sarilumab	-	200 mg q2w	-	150 mg/1.14 mL or 200 mg/1.14 mL solution in single-dose prefilled syringe or prefilled pen
IBD	Ustekinumab	Induction: < 55 kg: 260 mg > 55 kg to 85 kg: 390 mg > 85 kg: 520 mg Maintenance: 90 mg 8 weeks after the initial dose, then q8w (SC)	90 mg 8 weeks after the initial IV induction dose, then q8w	130 mg/26 mL solution in single-dose vial	45 mg/0.5 mL or 90 mg/mL solution in single-dose prefilled syringe 45 mg/0.5 mL in single-dose vial
	Vedolizumab	300 mg at weeks 0, 2 and 6, then q8w	-^c^	300 mg of lyophilized powder in single-use 20 mL vial	-^c^
	Natalizumab	300 mg q4w	-^d^	300 mg/15 mL solution in single-dose vial	-^d^
RA & IBD	Infliximab	*RA*: 3 mg/kg at weeks 0, 2 and 6, then q8w (may be increased to 10 mg/kg q8w or to dosing frequency of q4w) *CD*: 5 mg/kg at weeks 0, 2 and 6, then q8w (may be increased to 10 mg/kg q8w if loss of response) *UC*: 5 mg/kg at weeks 0, 2 and 6, then q8w	-^e^	100 mg of lyophilized powder in single-dose vial	-^e^
	Adalimumab	-	*RA*: 40 mg q2w (some patients not receiving methotrexate may benefit from dose increase to 40 mg q1w or 80 mg q2w) *CD*: 160 mg on day 1 (given in one day or split over two consecutive days); 80 mg on day 15 and 40 mg q2w starting on Day 29 *UC*: 160 mg on day 1 (given in one day or split over two consecutive days); 80 mg on day 15 and 40 mg q2w starting on Day 29	-	80 mg/0.8 mL, 40 mg/0.8 mL, and 40 mg/0.4 mL in single-dose prefilled pen 80 mg/0.8 mL, 40 mg/0.8 mL, 40 mg/0.4 mL, 20 mg/0.4 mL, 20 mg/0.2 mL, 10 mg/0.2 mL, 10 mg/0.1 mL in single-dose prefilled glass syringe 40 mg/0.8 mL in single-dose glass vial for institutional use only
	Golimumab	RA: 2 mg/kg at weeks 0 and 4, then q8w	*RA*: 50 mg q1m *UC*: 200 mg at week 0, 100 mg at week 2 and then 100 mg q4w	50 mg/4 mL solution in single-dose vial	50 mg/0.5 mL in single-dose prefilled syringe or single-dose prefilled autoinjector 100 mg/1.0 mL in single-dose prefilled syringe or single-dose prefilled autoinjector
	Certolizumab pegol	-	*RA*: 400 mg initially and at weeks 2 and 4, followed by 200 mg q2w; for maintenance dosing, 400 mg q4w can be considered *CD*: 400 mg initially and at weeks 2 and 4. If response occurs, 400 mg q4w	-	200 mg lyophilized powder in single-dose vial 200 mg/mL solution in single-dose prefilled syringe

*CD* Crohn’s disease, *IV* intravenous, *mg* milligram, *mL* milliliter, *qXd* every X day, *qXm* every X month, *qXw* every X week, *RA* rheumatoid arthritis, *SC* subcutaneous, *UC* ulcerative colitis

^a^This table lists FDA-approved products; deviations with EMA-approved product presentations or other indications are indicated as a footnote

^b^A SC formulation for rituximab is approved in oncology indications

^c^A SC formulation for vedolizumab is approved in IBD in the EU

^d^A SC formulation for natalizumab is approved in MS in the EU

^e^Biosimilar infliximab (Remsima®) is approved for subcutaneous administration in the EU

Prominent customer-centric product optimizations in disease area archetype 1 include changing the composition of the subcutaneous formulation for adalimumab. Accounting for "patient-reported pain" immediately following injection, the manufacturer changed the chemical buffer to help stabilize and preserve the mAb. This seemingly small change was shown to result in a reduction of pain at the injection site and ultimately in a significantly improved adherence and time on treatment overall (Bergman et al. 2020; Patel and Luu 2020). The finding is all the more important as decreased persistence to anti-tumor necrosis factor therapy had been reported to be associated with poorer clinical outcomes (Bluett et al. 2015). Additional product optimizations introduced for branded adalimumab comprise a reduced injection volume with a higher concentrated dosing solution, higher needle gauge, or modifications in the material of the injection devices (St Clair-Jones et al. 2020).

With the aim to optimize experience with the dosing procedure, to reduce the fear associated with needle use, and to aid people with impaired dexterity, in 2016, certolizumab pegol’s product presentations were complemented with a button-free autoinjector characterized by a wide, non-slip grip (Bailey et al. 2020). Supported with adequate training, the device could improve user confidence and satisfaction with subcutaneous self-administration. The introduction of a mini cartridge to be applied by means of a reusable autoinjector for the biologic etanercept in 2017 (Collier et al. 2017, Sedo 2018) may in the future also serve as a platform for mAbs. The product also utilizes an improved dosing solution that had been shown to lessen injection site pain as compared to the previous formulation (Cohen et al. 2019). Preference assessments comparing the novel reusable with the existing disposable automated pen device revealed perceived advantages for both injection aids, thus giving users the choice between two devices according to personal priorities (Collier et al. 2017).

To further facilitate compliance with at-home dosing, companies are implementing so-called patient support programs and app-based assistance tools including customized dosing reminders or injection and symptom trackers (Graigner et al. 2017, Lambrecht et al. 2021). Branded certolizumab pegol offers the option to apply the first partially reusable electromechanical injection device “of its kind available for use with biologic treatment in rheumatology and dermatology in Europe” (UCB 2021). Device design was actually guided by intended user feedback through human factor evaluations (Domańska et al. 2018). The injector was found to be preferred due to its ease-of-use over other subcutaneous devices in a study with certolizumab pegol-treated people from the Netherlands, Denmark, and Sweden (Pouls et al. 2020).

Manufacturers of follow-on biologics for mAbs in RA and IBD focus efforts on either using established injection device platforms or on customizing technologies to differentiate their products via unique, distinctive drug delivery characteristics. As for the branded counterparts, user and healthcare provider satisfaction and usability studies are part of the autoinjector development and commercialization strategy (Thakur et al. 2016; Tischer and Mehl 2018; Fleischmann et al. 2022). This iterative co-creation with customers is particularly important in disease areas in which people report problems with manual dexterity, pain linked to joint swelling in the hands, and general challenges with the self-injection procedure (Keininger and Coteur 2011).

Celltrion’s infliximab biosimilar received European Medicines Agency (EMA) approval for a subcutaneous dosing alternative in 2019 and FDA review is anticipated to be completed as a next milestone (Rose 2021; Verma et al. 2021), while branded infliximab is only available with an intravenous infusion regimen. This subcutaneous self-administered infliximab product presentation paired with telemedicine support and increasingly available RWD is suggested to lessen the time spent for travel and hospital attendance during dosing days and as such to reduce the pressure on healthcare systems (Ahmed et al. 2021; Perry and Jang 2020; Schreiber et al. 2022).

In aspiring to relieve the burden of parenteral dosing, the feasibility of oral dosing of mAbs is being examined (Philippart et al. 2016; New 2020Abramson et al. 2022). Different to injectable dosage forms, the individual dose level that can be administered orally is markedly reduced due to limited fill volumes of ingested oral dosage forms. Consequently, this approach is particularly interesting for mAbs in immunology, as inherent to their comparatively low-dose levels, a practicable oral dosing frequency may be achievable.

Different oral delivery technologies have recently advanced to clinical investigational stage. The first approach aims at precise delivery of biotherapeutics to gastrointestinal tissue thus avoiding high systemic exposure and potentially associated side effects. Here, biosimilar infliximab is being assessed for the feasibility of an oral version in the treatment of IBD. The aim is to target release in the colon and to protect the mAb from digestion in the stomach and upper gastrointestinal tract through local stabilization against proteases (Intract Pharma 2022). A second advanced oral delivery approach utilizes an orally ingestible robotic pill that auto-injects the biotherapeutic into the wall of the small intestine (Dhalla et al. 2022). The authors report that in an initial clinical trial with octreotide in healthy participants, administration of the pill was safe, well-tolerated, and yielded in an oral bioavailability of 65%. Assuming the scientific concept is confirmed in larger clinical trials and treatment can be realized at commercializable dose levels and dosing regimens, this approach has the potential to provide new clinical strategies in the future (Zhang et al. 2021).

### Disease area archetype 2—market with a small number of mAbs established for in-clinic or self-administration and no corresponding biosimilars available

MS, the second disease area archetype, is characterized by established non-mAb biological disease-modifying treatments available for subcutaneous self-administration. The majority of mAbs is offered with an intravenous infusion regimen, but the first subcutaneous mAb for self-administration has recently reached the market. To date, no biosimilar mAb is available in the US (United States Food and Drug Administration 2022c).

More precisely, subcutaneous self-administration with interferon beta (IFNβ) indicated to treat relapsing forms of MS is an established standard in the field (Kieseier 2011; Filipi and Jack 2020). Back in 1993, the first IFNβ was approved in the US and since then several others have become available (Bayas and Gold 2003). Due to their fixed dosing regimens and low injection volumes, IFNβ products are available in ready-to-use prefilled syringes and autoinjectors including devices with electronic adherence aids (Limmroth et al. 2017). IFNβ drug administration regimens range from every second day to every second week for the pegylated version that was authorized by the FDA in 2014 (Dashputre et al. 2017). In a German real-world study from 2021, this less frequent dosing alternative showed markedly higher scores for treatment satisfaction and convenience compared with previous therapies that included other IFNβ treatments (Menge et al. 2021).

The first mAb, natalizumab, an α4 integrin antagonist, entered the MS market in in 2004 with a fixed dose infused intravenously every 4 weeks over 1 h, and by now is available for the treatment of clinically isolated syndrome (CIS), relapsing–remitting MS (RRMS), and active secondary progressive MS (SPMS) (Rudick et al. 2013, HPI Tysabri®: United Stated Food and Drug Administration 2022a). While people with prior use of subcutaneous interferon regimens commonly value the option for self-administration, especially when facilitated with automated injection devices (Lugaresi et al. 2012), the improved efficacy of natalizumab over IFNβ therapy (Rudick and Panzara 2008; Lanzillo et al. 2012) is considered to outweigh the convenience disadvantage of more invasive intravenous dosing.

In 2014, the FDA approved alemtuzumab, an anti-cluster of differentiation 52 (CD52) mAb, for the treatment of RRMS (Ruck et al. 2015). The medicine is available with a fixed-dose intravenous regimen for two treatment courses. During the first treatment course, alemtuzumab is administered over 4 h on five consecutive days and on three consecutive days during the second treatment course 12 months later. Additional treatment courses may be considered with drug administrations of three consecutive days (HPI Lemtrada®: United Stated Food and Drug Administration 2022a). This comparatively convenient infrequent dosing regimen is to some extent counterbalanced by the need for regularly monitoring the increased risk of autoimmunity (Garnock-Jones 2014). While alemtuzumab when delivered via the subcutaneous route may reduce infusion-related adverse events as compared to intravenous dosing (Perumal 2012), a subcutaneous formulation is not available for use in MS.

The anti-CD20 mAb ocrelizumab was first authorized in the US in 2017 (Frampton 2017) and by now is applied for the treatment of relapsing forms of MS (RMS), including CIS, RRMS, PPMS, and for the treatment of SPMS (Stahnke et al. 2018, Weinstock-Guttman et al. 2022, HPI Ocrevus®: United Stated Food and Drug Administration 2022a). The mAb was introduced with an intravenous fixed-dose regimen. Here, the initial treatment cycle comprises two separate infusions on days 1 and 15, respectively, followed by twice yearly maintenance doses. The United Kingdom’s (UK) National Institute for Health and Care Excellence (NICE) noted in their final appraisal document on “Ocrelizumab for treating relapsing–remitting multiple sclerosis” that based on insights from “patient experts” “patients would value a treatment with less frequent dosing or monitoring,” acknowledging that the intervention is less interruptive for people’s lives compared to other treatments (National Institute for Health and Care Excellence 2018).

To optimize satisfaction and quality of life with intravenous mAb treatments and to improve healthcare institutional resource utilization in MS, efforts are made to support home-based and outpatient infusion management (Vijayan et al. 2017; Schultz et al. 2021; Barrera et al. 2022; Räuber et al. 2022). It was found that people are generally open to receiving the intravenous treatment at home and that supporting health services need to ensure safety and be efficient, responsive, and flexible. Thus, health services should also allow for administering the medication at individually preferred times during the day (Rath et al. 2021). Supporting measures include designing appropriate home health care services for natalizumab or shortening ocrelizumab’s intravenous infusion time from 3.5 to 2 h (Schultz et al. 2019; Hartung et al. 2020).

In 2020, a second anti-CD20 mAb, ofatumumab, was authorized by the FDA for the treatment of CIS, RRMS, and active SPMS (HPI Kesimpta®: United Stated Food and Drug Administration 2022a). Notably, the mAb was directly introduced with a subcutaneous formulation for self-administration, a fixed dose, and a dosing volume of 0.4 mL. Using an existing autoinjector platform that was previously applied to other products of the same manufacturer (HPIs Cosyntex®, Elrezi®, Hyrimoz®: United Stated Food and Drug Administration 2022a), ofatumumab was launched both in a prefilled syringe and in an automated pen injector. In its final appraisal document on “Ofatumumab for treating relapsing multiple sclerosis,” the UK’s NICE notes that they heard from “patient experts” “that a treatment that could be self-administered monthly is less disruptive to people’s lives than treatments administered by intravenous infusions in hospital, so would be valued by people with multiple sclerosis” (National Institute for Health and Care Excellence 2021). Launching a mAb in two different presentations accounts for distinct preferences (Kivitz et al. 2018; Vermeire et al. 2018) already at first introduction of the novel medicine. Additionally, a manufacturer-initiated study revealed user and nurse preference for the autoinjector over their current injectables mainly due to the “ease to perform self-injection with the pen” and “patient able to use independently” (Ross et al. 2021).

In 2021, a subcutaneous version of natalizumab with an overall shorter infusion time as compared to the intravenous regimen received marketing authorization in the EU (Summary of Product Characteristics (SMPC) Tysabri®: European Medicines Agency 2021, López et al. 2021). The product is available in prefilled syringes, two of which need to be administered at each dosing day with a monthly dosing regimen; home treatment is not recommended. In the same year, the manufacturer did receive a complete response letter (CRL) from the FDA to their supplemental Biologic License Application for the subcutaneous dosing alternative (BioSpace 2021); the underlying reasons for the CRL are unknown to the author of this review. Also, a subcutaneous dosing alternative in development for ocrelizumab has reached Phase 3 clinical development stage (clinicaltrials.gov 2022). Table 3 summarizes the mAb presentations authorized for the treatment of adult people diagnosed with MS.

**Table 3:** mAbs authorized for the treatment of MS in the US (up until September 2022): administration routes, dosing regimens and product presentations (adult indications)

**mAb**	**Administration route and dosing regimen**^a^		**IV product presentations**^a^	**SC product presentations**^a^
	**IV**	**SC**		
Natalizumab	300 mg q4w	-^b^	300 mg/15 mL in single-dose vial	-^b^
Alemtuzumab	Initial treatment (2 courses): First course of 12 mg/day on 5 consecutive days; second course of 12 mg/day on 3 consecutive days 12 months after first treatment course Subsequent treatment courses: 12 mg/day on 3 consecutive days as needed, at least 12 months after the previous course	-	12 mg/1.2 mL in single-dose vial	-
Ocrelizumab	Start dose: 2*300 mg separated by two weeks Subsequent doses: 600 mg q6m	-	300 mg/10 mL in single-dose vial	
Ofatumumab	-	Initial dosing: 20 mg administered at weeks 0, 1, and 2 Subsequent dosing: 20 mg q1m starting at week 4	-	20 mg/0.4 mL solution in single-dose prefilled pen or single-dose prefilled syringe

*IV* intravenous, *mg* milligram, *mL* milliliter, *qXd* every X day, *qXm* every X month, *qXw* every X week, *SC* subcutaneous

^a^This table lists FDA-approved products; deviations with EMA-approved product presentations are indicated as a footnote

^b^A SC formulation for natalizumab in MS is approved in the EU

### Disease area archetype 3—market with variety of established mAb treatments for healthcare provider administration and a number of corresponding biosimilars available

The ONC area represents the third selected disease area archetype. Here, mAbs for the treatment of malignancies have been on the market for decades, but due to at times severe IRRs and frequently high individual mAb dose levels, products are not yet available for self-administration. Until recently, due to the lack of technologies that facilitate high-dose subcutaneous administration, mAb products were offered as intravenous infusions only. Today, a number of subcutaneous dosing alternatives have been established. The first biosimilar mAbs have been authorized, currently with intravenous dosing regimens only.

More specifically, since the approval of rituximab for the treatment of B-cell malignancies back in 1997 (Pierpont et al. 2018), numerous other mAbs have become available and represent an important modality in the treatment of cancer (Zahavi and Weiner 2020). The large majority of these mAbs are authorized for intravenous administration and need to be administered by a healthcare professional (Kafatos et al. 2020). Depending on the nature and severity of IRRs, in some instances patients have to be monitored closely to provide medical treatment when required (HPIs Rituxan®, Erbitux®: United Stated Food and Drug Administration 2022a, Graham 2009). Understandably, these significant drug administration efforts add to the already high expenditures for mAb treatments overall (Chadda et al. 2013).

As described for intravenous treatments in MS, also in cancer care, rapid infusion regimens (Atay et al. 2012, Gozzetti et al. 2020) or less frequent dosing regimens (Lala et al. 2020) represent an attempt to reduce the expenditures associated with drug administrations as well as the time people treated with mAbs have to spend in the clinic. Here, the pandemic has intensified the elaboration of clinical strategies for optimizing infusion center care (Hanna et al. 2021). The organization of home health services is a pivotal step to reduce time and traveling expenditures associated with in-clinic dosing. Notably, efforts are mandated to ensure that costs underlying provider work supporting at-home dosing and monitoring efforts remain within an affordable range (Franken et al. 2020).

Initially, mAbs in ONC were made available with a body weight- or body surface area-adjusted dosing regimen (Hendrikx et al. 2017); an approach that was based on the way cytotoxic agents with a narrow therapeutic window are being administered (Egorin 2003). With the increasing understanding of the pharmacokinetic-pharmacodynamic and -safety correlation (Paci et al. 2020), attempts are made to either develop mAbs with a fixed dosing regimen from the very beginning (Garg et al. 2014) or to change from body size-based dosing to fixed dosing as a lifecycle management activity following initial launch (Freshwater et al. 2017; Bei et al. 2020).

The subsequent step towards more customer-friendly drug delivery of mAbs in ONC represented the development of subcutaneous dosing options for mAbs (Bittner and Schmidt 2012). Immanent to the at times high individual dose levels, compared to mAbs in immunology for example (refer to disease area archetype 1), developing subcutaneous injection regimens was initially complicated due to a number of technical challenges. With the introduction of methodologies to achieve high-concentration solutions (Mahler et al. 2009; Jiskoot et al. 2022) and the co-administration of the dispersion enhancer hyaluronidase (Frost 2007), the first moves were made to reduce the overall dosing volume and to facilitate spreading of an injected fluid in the interstitial space.

Approved high-volume subcutaneous treatments that apply these technologies can maintain the infrequent dosing regimen of the initially marketed intravenous presentations. Up until September 2022, the subcutaneous administration alternatives for rituximab in B-cell malignancies (11.7 and 13.4 mL; FDA approval in 2017), trastuzumab in HER2-positive early and metastatic breast cancer (5 mL, FDA approval in 2019), and daratumumab in multiple myeloma (15 mL; FDA approval in 2020) have been authorized in the US (Yelvington 2018, Center for Drug Evaluation and Research 2020, Duco 2020, Kading and Beck 2021). These subcutaneous mAb presentations are all available with fixed-dose regimens omitting the need for body size-adjusted dose calculation. Dosing solutions are offered in vial presentations and are injected manually by a healthcare provider using a handheld syringe or an infusion set.

To simplify administration of subcutaneous trastuzumab, a ready-to-use on-body delivery system that is attached to the skin via an adhesive plaster had been developed (Bittner et al. 2012, Gligorov 2022). A small study in 102 participants diagnosed with HER2-positive early breast cancer revealed that subcutaneous at-home injections by a healthcare professional did not introduce new safety signals and respondents agreed that they had benefit from at-home administration to a large (22%) or very large extent (78%) (Denys et al. 2020). Time-and-motion and preference assessments demonstrated user preferences of subcutaneous over intravenous dosing in a healthcare institutional setting, regardless of on-body delivery system or handheld syringe delivery (Pivot er al 2014). As trastuzumab is not permitted for home- or self-administration, the device was not commercialized at the time of marketing authorization of the subcutaneous trastuzumab formulation in the EU back in 2013.

The aspect that mAbs are increasingly developed for combination therapy (Henricks et al. 2015; Peterson et al. 2018), a condition that further adds to the complexity of parenteral dosing, makes ONC an intriguing disease area archetype from a drug delivery perspective. Consequently, manufacturers started co-formulating two mAbs within the same dosing vehicle as a fixed-dose combination. The first fixed-dose combination of two mAbs included pertuzumab and trastuzumab and is indicated for the treatment of people diagnosed with with HER2-positive early and metastatic breast cancer. The medication is available with a subcutaneous dosing regimen and has been approved by the FDA in 2020 (Gao et al. 2021). It had been shown that patients strongly preferred this fixed-dose combination over sequential intravenous infusion of the individual mAbs in separate formulations (O'Shaughnessy et al. 2021). Remarkably, when approving Phesgo, in its press release, the FDA specifically highlights that “…Phesgo offers an out-patient option for patients…” (United States Food and Drug Administration 2020), an aspect that is considered very relevant especially in times of the coronavirus pandemic. The first fixed-dose combination of two immunotherapy mAbs, the programmed death receptor-1 inhibitor nivolumab and the lymphocyte activation gene-3 blocking antibody relatlimab, received FDA approval for the treatment of unresectable or metastatic melanoma in 2022 (HPI OpdualagTM: United Stated Food and Drug Administration 2022a). The formulation is administered as a fixed-dose intravenous infusion regimen.

Table 4 summarizes the high-dose subcutaneous single-active mAb formulations and fixed-dose combinations authorized for the treatment oncological indications in the US.

**Table 4:** High-dose subcutaneous single-active mAb formulations and fixed-dose combinations authorized for the treatment oncological indications in the US (up until September 2022): administration routes, dosing regimens and product presentations

**mAb**	**Administration route and dosing regimen**^a^		**IV product presentations**^a^	**SC product presentations**^a^
	**IV**	**SC**		
Trastuzumab	*Adjuvant HER2* + *BC*: Loading dose: 4 mg/kg Maintenance dose: 2 mg/kg q1w for 12 weeks followed by 6 mg/kg q3w to complete a total of 52 weeks *or* Loading dose: 8 mg/kg Maintenance dose: 6 mg/kg q3w for 52 weeks *HER2* + *mBC*: Loading dose: 4 mg/kg Maintenance dose: 2 mg/kg q1w *HER2* + *GC*: Loading dose: 8 mg/kg Maintenance dose: 6 mg/kg q3w	*HER2* + *BC*: 600 mg trastuzumab and 10,000 units hyaluronidase q3w	150 mg lyophilized powder in single-dose vial 420 mg lyophilized powder in single-dose vial	600 mg trastuzumab and 10,000 units hyaluronidase per 5 mL solution in single-dose vial
Rituximab	*NHL*: 375 mg/m^b^ (indication-dependent schedule) *CLL*: Cycle 1: 375 mg/m^b^ Cycles 2 − 6: 500 mg/m^b^ q28d	The first dose is administered IV *NHL*: 1400 mg rituximab and 23,400 units hyaluronidase (indication-dependent schedule) *CLL*: 1600 mg rituximab and 26,800 units hyaluronidase (indication-dependent schedule)	100 mg/10 mL solution in single-dose vial 500 mg/50 mL solution in single-dose vial	1400 mg rituximab and 23,400 units hyaluronidase human per 11.7 mL solution in single-dose vial 1600 mg rituximab and 26,800 units hyaluronidase human per 13.4 mL solution in single-dose vial
Daratumumab	*MM*: 16 mg/kg; may be split over two consecutive days with 8 mg/kg on day 1 and day 2 (indication-dependent schedule)	*MM*: 1800 mg daratumumab and 30,000 units hyaluronidase (indication-dependent schedule)	100 mg/5 mL solution in single-dose vial 400 mg/20 mL solution in single-dose vial	1800 mg daratumumab and 30,000 units hyaluronidase per 15 mL solution in single-dose vial
Pertuzumab	*Adjuvant HER2* + *BC*: Loading dose: 840 mg Maintenance dose: 420 mg q3w (combination with trastuzumab IV or SC) for up to 18 cycles *Neo-adjuvant HER2* + *BC*: Loading dose: 840 mg Maintenance dose: 420 mg q3w (combination with trastuzumab IV or SC) for 3 to 6 cycles *HER2* + *mBC*: Loading dose: 840 mg Maintenance dose: 420 mg q3w	-	420 mg/14 mL in single-dose vial	-
Pertuzumab + trastuzumab FDC	For single-active formulations see above	*Adjuvant HER2* + *BC*: Loading dose: 1200 mg pertuzumab, 600 mg trastuzumab, and 30,000 units hyaluronidase Maintenance dose: 600 mg pertuzumab, 600 mg trastuzumab, and 20,000 units hyaluronidase for up to 18 cycles *Neo-adjuvant HER2* + *BC*: Loading dose: 1200 mg pertuzumab, 600 mg trastuzumab, and 30,000 units hyaluronidase Maintenance dose: 600 mg pertuzumab, 600 mg trastuzumab, and 20,000 units hyaluronidase for 3 to 6 cycles *HER2* + *mBC*: Loading dose: 1200 mg pertuzumab, 600 mg trastuzumab, and 30,000 units hyaluronidase Maintenance dose: 600 mg pertuzumab, 600 mg trastuzumab, and 20,000 units hyaluronidase	For single-active formulations see above	1200 mg pertuzumab, 600 mg trastuzumab, and 30,000 units hyaluronidase/15 mL in single-dose vial 600 mg pertuzumab, 600 mg trastuzumab, and 20,000 units hyaluronidase/10 mL in single-dose vial
Nivolumab	*Various cancer indications*: Indication-dependent dose and schedule	-	40 mg/4 mL, 100 mg/10 mL, 120 mg/12 mL, and 240 mg/24 mL solution in single-dose vial	-
Relatlimab^b^	-	-	-	-
Nivolumab + relatlimab FDC	*UMM*: 480 mg nivolumab and 160 mg relatlimab q4w (adverse event-dependent dose modifications)	-	240 mg of nivolumab and 80 mg of relatlimab per 20 mL in single-dose vial	-

*CLL *chronic lymphocytic leukemia, *FDC* fixed-dose combination, *HER2* + *BC* HER2-overexpressing breast cancer, *HER2* + *GC* HER2-overexpressing gastric cancer, *HER2* + *mBC* metastatic, *HER2* + *BC* HER2-overexpressing breast cancer, *IV*intravenous, *mg* milligram, *mL* milliliter, *MM* multiple myeloma, *NHL* non-Hodgkin’s lymphoma, *qXd* every X day, *qXm*, every X month, *qXw* every X week, *SC* subcutaneous, *UMM* unresectable or metastatic melanoma

^a^This table lists FDA-approved products

^b^Relatlimab is only available in the FDC with nivolumab

The initial follow-on biologics for mAbs in ONC indications have been approved by the FDA (Galvão 2020). Not only have manufacturers mimicked the originator medications, in some cases they also optimized the product presentation to make it more user-friendly. Improvements include extending the in-use stability to mitigate the impact of cold-chain rupture and exceptional temperature excursions on drug wastage and the quality of the product (Vieillard et al. 2017; Park et al. 2020). Even without the implementation of product optimizations, biosimilars are considered a customer-centric alternative to their branded counterparts solely based on the potential to increase access to mAb-based cancer medicines globally via reduced product costs compared to the originator mAb (Patel et al. 2018; Shelbaya et al. 2021). Here, interestingly, actual realization of a switch from branded to follow-on biologic or a switch from one to another biosimilar varies significantly from country to country. Regional differences, such as prescriber and/or patient insecurity concerning efficacy and safety, conservative prescribing patterns, reimbursements and billing policies, supply logistics, and legal considerations have been suggested as limiting factors to broader adoption of biosimilars (Cortes et al. 2020; Azuz et al. 2021).

## Discussion

The term “customer centricity,” indicating that fulfilling customer demands is as important as creating the product or services themselves (Ceesay 2020), is not new and applied across various industries. It has, however, gained increasing attention in healthcare facing high economic pressure, especially in light of the coronavirus pandemic. Here, customer centricity aims at developing convenient medicines that are globally affordable for people, providers, and the healthcare system as whole. For biotherapeutics, such as mAbs, customer-centric product offerings and treatment management concepts ideally facilitate a flexible care setting and thus allow for drug administration and treatment monitoring outside of a controlled healthcare institutional environment. Efforts in the field go beyond the described improvements of the drug delivery profile and product presentation and are in many cases driven by pharmaceutical scientists from different disciplines.

The realization of product optimizations differs across the distinctive disease area archetypes and depends on customer and market needs as well as on the clinical and technical feasibility of the intervention. In an indication in which the identification of disease-modifying medicines represents a major unmet need, initial drug delivery efforts focus on enabling treatment per se. This is for example the case for mAbs in ONC where any new and promising molecule is developed with the aim to offer it to people diagnosed with a given malignancy as soon as feasible. Equally, for mAbs with demonstrated efficacy but unfavorable exposure-related safety findings, product optimizations target a reduction in the incidence and severity of adverse events through lowering post-infusion serum levels. This may be achieved for example by increasing the dosing frequency (Bai et al. 2012), a schedule change that reduces convenience and increases healthcare institutional burden associated with shorter dosing intervals.

**Fig. 3:**
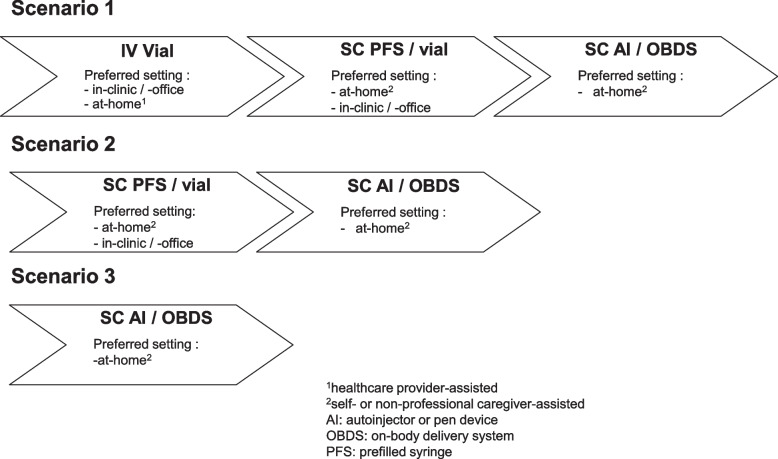
Possible launch scenarios for intravenous versus subcutaneous dosing regimens for mAbs

As per the definition in this review article, the journey to more customer-centric products starts with the availability of an efficacious product with an acceptable risk/benefit ratio. Across the selected disease archetypes, the existence of established drug delivery technology platforms plays a pivotal role in enabling customer-centric product presentations early on, ideally already at initial launch of the mAb. Combining the learnings from these distinct disease area archetypes, Fig. 3 illustrates possible launch scenarios for intravenous and subcutaneous dosing regimens for mAbs. The underlying assumption is that mAbs with dosing volumes of up to approximately 2 mL are conventionally dosed in prefilled syringes and automated pen or autoinjector devices, while higher volume mAbs are provided in vial presentations and possibly in the future in larger automated pen and autoinjector devices or in automated on-body delivery systems.

In scenario 1, at initial molecule launch, the mAb is available with an intravenous dosing regimen for in-clinic, in-office, or healthcare provider-supervised at-home administration. As a subsequent lifecycle management, a subcutaneous dosing alternative is becoming available, either in a prefilled syringe for self-administration or in a vial presentation for manual or infusion pump-assisted injection by a caregiver. Product presentations vary from case to case depending on the dosing volume and overall feasibility of a stable liquid solution. Most frequently, medications are offered with a fixed dose, unless a body size-, safety-, or response-dependent regimen is justified (Strik et al. 2018). In a third step, the manufacturer introduces automated injection devices. This scenario was for example realized for tocilizumab in RA. Initially approved in 2010 with a vial for intravenous infusion and a monthly regimen (HPI Actemra®: United Stated Food and Drug Administration 2022a), the subcutaneous dosing alternative was made available sequentially with a prefilled syringe in 2013 (Burmester et al. 2014, Shetty et al. 2014), followed by an autoinjector device as a lifecycle management in 2018 (Genentech 2013). To keep the injection volume low and account for readily available devices at the time, the subcutaneous dosing frequency was increased to weekly and every 2 weeks with a dosing volume of 0.9 mL (Fettner et al. 2019).

Scenario 1 also applies for mAbs in the treatment of malignancies. While intravenously dosed mAbs are an established treatment modality, development of subcutaneous dosing alternatives started only years after the first mAb approval (Salar et al. 2014; Jackisch et al. 2019). This is due to at times severe and even fatal IRRs and comparatively high individual dose levels that challenged the development of convenient subcutaneous dosing regimens. With increasing knowledge about the general feasibility of high-volume subcutaneous dosing, advances in high-concentration formulations and the co-administration of the dispersion enhancer hyaluronidase, by now, this route of administration has become a key focus area of drug delivery scientists across indications (Bookbinder et al. 2006; Mathaes et al. 2016). While in ONC, mAb dosing in the clinic is standard practice, today, efforts in disease management support are made to shift treatment and monitoring outside of the clinic (Denys et al. 2020). Independent at-home administration for high-dose mAbs has not yet been realized, but represents a thinkable option in the future with the advancement of larger volume on-body delivery systems (Bittner and Schmidt 2021).

Notably, next to a lack of technical and clinical feasibility, it is also the healthcare provider reimbursement model applied in a given legislation that challenges subcutaneous administration in a decentralized setting. Roughly speaking, one can discriminate between fee-for-service payment models with separate service-specific payments and models where a medical provider receives a predetermined payment for a sequence of related healthcare services (Einav et al. 2022). From a drug delivery perspective, whereas the first model incentivizes the complexity and quantity of care and as such incentivizes more complex intravenous infusions, the latter rewards the quality of care and thus ready-to-use subcutaneous regimens with the potential for at-home administration. Here, any efforts that facilitate shifting treatment outside of the clinic are usually valued.

In launch sequence scenario 2, the mAb enters the market directly with a subcutaneous formulation. Currently, the prerequisite for this approach is that doses are low enough to apply established formulation and device technologies. Products are offered either in a prefilled syringe or vial configuration. Subsequently, upon availability, automated injection devices are introduced as a lifecycle management. This scenario is depicted in how a number of mAb products were introduced into the RA and IBD indications. The sequential market introduction of increasingly optimized product presentations did allow manufacturers to consider user feedback on the selection of a ready-to-use device. The device portfolio was subsequently expanded with additional product offerings for people who prefer one over another injection aid. Examples would be branded golimumab or adalimumab. Golimumab was first approved in the US with a prefilled syringe in 2009 (HPI Simponi®: United Stated Food and Drug Administration 2022a), followed by the introduction of an autoinjector 4 years later in 2013 (Center for Drug Evaluation and Research 2013). This device was favorably evaluated in a prospective study in biologic-naïve people with active RA (Schulze-Koops et al. 2013). Likewise, adalimumab was first authorized with a prefilled syringe specifically designed for self-administration for people with stiffness in their hands due to destructive progression of RA as well as with a vial for institutional use in 2002 (HPI Humira®: United Stated Food and Drug Administration 2022a). Approval of the automated pen device followed sequentially in 2006 (BioSpace 2006). A comparison of the pen device with the established prefilled syringe as assessed in a Phase 2 trial in participants diagnosed with RA revealed preference for the automated injector based on its perceived ease of use and resulting convenience (Kivitz et al. 2006).

The framework underlying scenario 3, where mAbs are directly and solely launched with a subcutaneous dosing regimen presented in ready-to-use automated injectable presentations, to date comprises mAbs and their follow-on biologics with low dosing volumes that are generally well tolerated. These favorable features enable the use of established drug delivery platforms. Currently, this scenario is being realized for adalimumab follow-on biologics (Ghil et al. 2019, HPIs Hulio®, Hadlima: United Stated Food and Drug Administration 2022a). Another example for scenario 3 is the introduction of ofatumumab in MS, where the mAb was directly obtainable with both a prefilled syringe and an automated injection pen. Leveraging an established autoinjector platform, the manufacturer conducted the pivotal Phase 3 study for ofatumumab with a prefilled syringe and bridged to the autoinjector in a Phase 2 trial that demonstrated bioequivalence of ofatumumab administered by the autoinjector versus the prefilled syringe (Bar-Or et al. 2022).

A comparison of activities among biosimilar manufacturers qualifying as customer-centric as defined in this article did reveal different focus areas across the designated disease area archetypes. Especially in the event of more than one follow-on biologic accessible for the same originator, the competition for market shares via a customized product profile is expected to increasingly gain momentum. With this, the wider range of injection devices qualified will contribute to fulfilling the needs of a larger user population. Here, the application of established technology platforms for a variety of medications accounts for both, the familiarity of prescribers with the device as well as learnings from challenges associated with their application and how to most appropriately overcome these. For mAbs predominantly available for in-clinic mAb administration, the described seemingly smaller product changes, such as a change from a lyophilized powder for reconstitution to a ready-to-administer liquid formulation or an increased storage time and shelf-life can be an advantage for one over another biosimilar. This is due to for instance improved distribution and handling logistics, reduced drug wastage or more economical resource utilization in the clinic (Smale et al. 2021). It is of note in the context of customer-centric biosimilar offerings that depending on the country and associated pricing, reimbursement, and demand-side policies (Rémuzat et al. 2017), lower overall treatment costs per se may provide an access advantage over their originator counterpart (Kvien et al. 2022) without the need for further optimizing the product profile. In an attempt to facilitate access to treatment, the FDA has designated the first mAb, an adalimumab biosimilar, as interchangeable with the reference product in 2021 (United States Food and Drug Administration 2021), meaning that the biological product “may be substituted for the reference product without the involvement of the prescriber” (United States Food and Drug Administration 2017).

## Summary and outlook

Advancing customer-centric medicinal products is an adaptive process across the lifecycle of a mAb-based medicine that aims to address individual needs of people treated as well as those of the healthcare ecosystem as a whole. The actual realization of product optimizations is in turn influenced by the safety and efficacy profile of a medication, market maturity, and the availability of enabling technologies. Here, the application of platform technologies that have the potential to be utilized for mAbs across different indications offer the possibility to launch a novel mAb already with the most preferred drug delivery profile or even with a variety of different customized options at initial market authorization. This will be especially the case for disease areas in which mAb-based medicines currently are among the investigated targets, such as Alzheimer’s disease or rare diseases (Tambuyzer et al. 2020; Lacorte et al. 2022).

Subcutaneous at-home administration of low-volume mAb formulations has been feasible for decades and illustrates the long-standing efforts in the field. As a next pivotal step to also warrant high-volume subcutaneous home administration with dosing volumes exceeding 5 mL, on-body delivery systems need to leave the exploratory stage and require implementation into clinical practice. Electronic adherence aids that further engage people treated with mAbs and their care partners into disease management while still guaranteeing a remote contact with the physician should be implemented in parallel. A significant change in dosing paradigm for mAbs would be a shift from parenteral to oral administration, with a variety of technologies in early development. A key prerequisite here is that drug administration schedules can still be managed based on the mAb’s dose level and the cost of goods sold associated with the provision of these at times device-based technologies. Manufacturers need to partner with specialized biotechnology companies and, like with any innovation, need to afford some upfront investment at risk. This way oral delivery platforms may become a reality for mAbs across disease areas.

A field of increasing relevance is the sustainability of novel drug delivery technologies. Manufacturers will have to do their homework to understand whether for example reusable technologies indeed offer a more environmental dosing alternative, or whether possible advantages come with the challenge of reduced user-friendliness.

Pharmaceutical scientists are involved in product optimizations across different disciplines, that is, besides their role as practicing healthcare provider, in formulation and device development, nonclinical and clinical pharmacokinetics and pharmacology, or in regulatory affairs and market access. As such, we have the encouraging opportunity and mandate to leverage existing insights and synergies across different indications and thus avoid repeating assessments and reinventing development and commercialization pathways from the beginning. The work on improving mAb products should involve co-creation not only with customers, but also collaborations between academia, manufacturers, and biotechnology companies.

## Data Availability

Not applicable (review article).

## Abbreviations

CD: Crohn’s disease
CD52: Cluster of differentiation 52
CIS: Clinically isolated syndrome
CRL: Complete response letter
EMA: European Medicines Agency
EU: European Union
FCS: Flexible care setting
FDA: Food and Drug Administration
HPI: Highlights of Prescribing Information
IBD: Inflammatory bowel disease
IFNβ: Interferon beta
IRR: Infusion-related reaction
mAb: Monoclonal antibody
MS: Multiple sclerosis
NICE: National Institute for Health and Care Excellence
ONC: Oncology
RA: Rheumatoid arthritis
RMS: Relapsing forms of multiple sclerosis
RRMS: Relapsing-remitting multiple sclerosis
RWE: Real-world evidence
RWD: Real-world data
SMPC: Summary of Product Characteristics
SPMS: Secondary progressive multiple sclerosis
UC: Ulcerative colitis
UK: United Kingdom
US: United States
